# Cefiderocol-Based Regimen for Acinetobacter NDM-1 Outbreak

**DOI:** 10.3390/antibiotics13080770

**Published:** 2024-08-15

**Authors:** Giovanna Travi, Francesco Peracchi, Marco Merli, Noemi Lo Re, Elisa Matarazzo, Livia Tartaglione, Alessandra Bielli, Giorgia Casalicchio, Fulvio Crippa, Chiara S. Vismara, Massimo Puoti

**Affiliations:** 1 Division of Infectious Diseases, ASST Grande Ospedale Metropolitano Niguarda, 20162 Milan, Italy; giovanna.travi@ospedaleniguarda.it (G.T.); marco.merli@ospedaleniguarda.it (M.M.); fulvio.crippa@ospedaleniguarda.it (F.C.); massimo.puoti@ospedaleniguarda.it (M.P.); 2 School of Medicine and Surgery, University of Milano-Bicocca, 20854 Monza, Italy; 3 Clinical Microbiology Department, University Sacro Cuore, 00168 Rome, Italy; noemi.lore@ospedaleniguarda.it; 4 Division of Clinical Microbiology, ASST Grande Ospedale Metropolitano Niguarda, 20162 Milan, Italy; elisa.matarazzo@ospedaleniguarda.it (E.M.); livia.tartaglione@ospedaleniguarda.it (L.T.); alessandra.bielli@ospedaleniguarda.it (A.B.); giorgia.casalicchio@ospedaleniguarda.it (G.C.); chiara.vismara@ospedaleniguarda.it (C.S.V.)

**Keywords:** *Acinetobacter baumannii* complex, NDM, cefiderocol, colistin

## Abstract

Variable outcomes have been reported with cefiderocol in infections due to carbapenem-resistant *Acinetobacter baumannii* (CRAB). Nonetheless, it may be the only option for metallo-beta-lactamase-producing strains. We describe an outbreak of NDM-CRAB infections treated with cefiderocol. Thirty-eight patients were colonized and/or infected. Thirteen patients developed a systemic infection. A clinical cure was achieved in 10 (83%) patients, one VAP and 9 BSIs, at day 7. In vitro, the activity of cefiderocol does not appear to match in vivo effectiveness using currently available commercial tests. Despite high clinical cures, overall mortality remains high in severely ill patients. Cefiderocol may be considered in this specific setting, though the implementation of susceptibility tests and infection control measures is mandatory.

## 1. Introduction

*Acinetobacter calcoaceticus-baumannii complex* (ABC) is a Gram-negative non-fermenting bacillus formerly considered a commensal opportunist. It acquired growing relevance especially in critically ill patients and severe burns as a frequent cause of healthcare-associated infections, probably due to its ability to survive on surfaces and to resist disinfection and desiccation [1].

The capability of *Acinetobacter* to acquire resistance mechanisms resulted in an extensively drug-resistant phenotype and its diffusion in the healthcare setting, especially among subjects requiring mechanical ventilation and other invasive devices to sustain vital functions. Surface porins are frequently expressed, contributing to drug-resistant phenotypes [2].

Carbapenem resistance is usually mediated by the production of oxacillinases, such as OXA-24/40-like and OXA-23-like. Nonetheless, metallo-β-lactamases (MBL) and additional serine carbapenemase have also been reported. Bla_NDM_-type genes were found to be located on either plasmid or chromosome in *A. baumannii* and the identification of a composite transposon Tn125 in both *A. baumannii* and *Enterobacterales* suggested a role of *Acinetobacter* in NDM transmission and diffusion to other species [3]. NDM-1 (New Delhi metallo-beta-lactamase 1)-expressing ACB was isolated from environmental and clinical specimens in different countries [4,5,6,7].

Treatment options for extensive-drug-resistant *A. baumannii* are extremely limited. Carbapenem-resistant strains are often susceptible to polymyxins only, but their use is often avoided due to the high risk of nephrotoxicity, the limited clinical and microbiological efficacy, and the low diffusion in the epithelial lining fluid [8]. Current guidelines suggest the use of sulbactam as a first line treatment for carbapenem-resistant *A. baumannii* (CRAB), which have no activity against MBL-producing strains [9,10]. Neither the new combination of sulbactam/durlobactam represents an option in this setting [11]. Cefiderocol showed a lower success rate compared to the best available therapy against *A. baumannii* in the randomized controlled trial (CREDIBLE) [12], though observational and retrospective cohorts provided a higher clinical efficacy [13,14]. Nowadays, in CRAB infections, cefiderocol is one of the few therapeutic choices, particularly when wide resistance mechanisms, such as bla_NDM_, have been acquired.

On the other hand, surveillance and active testing are nowadays extremely important. As we know, ABC is highly resistant to the environment and may be found in multiple body sites [15].

Active surveillance cultures for asymptomatic colonization of CRAB are not routine clinical practice in all centers, especially in resource-limited healthcare settings [16]. The use of active surveillance rectal swabs, followed by isolation and contact precautions, seems to be inversely associated with diffusion of CRAB and consequently CRAB infection [17]. This consideration has been particularly true in the intensive care unit, where invasive procedures and immunosuppression enhanced the risk of infections [2]. There is no consensus on the sampling sites for CRAB isolation or on the use of a horizontal or vertical approach for screening during hospitalization [18]. Some studies suggested the skin has the highest rate in the detection of CRAB whereas some others revealed rectal swabs may to be positive in the highest number of colonized by CRAB if a single sample is collected [19].

Herein, we here report a case series of NDM-producing *A. baumannii* colonization and infections, with particular attention to cases of bloodstream infections treated with cefiderocol-based regimen.

## 2. Results

### 2.1. Demographic and Clinical Characteristics

Thirty-eight consecutive patients with colonization and/or infection sustained by NDM ABC were included. All patients were admitted to the intensive care unit (ICU) or Burns Center. Demographic and clinical characteristics are shown in Table 1. The median age was 68 (55–74) years old, the most common reasons for hospital admission were infections (36%), burns (18%), and major trauma (16%). Charlson comorbidity index was 4 (2–5), 14 (40%) patients receiving immunosuppressive therapy.

### 2.2. Colonization and Infection Course

Thirteen patients developed infection: 8 primary bloodstream infections (BSI), 3 central venous catheters (CVC) related bloodstream infections, 1 ventilation-associated pneumonia (VAP), and 1 osteomyelitis. Time from admission to first reported colonization was 15 (6–28) days, 19 (5–23) in the infected and 14 (5–31) in the colonized group (*p* = 0.445). The median time from the first evidence of colonization at surveillance swab to infection was 8 (7–13) days, while infection developed 21 (6–27) days after the hospital admission. Among colonized patients, 2 out of 7 patients who underwent abdominal surgery developed infection, while 5 out of 6 patients with severe burns (83%) experienced infection (*p* = 0.021). ICU stay lasted 21 (15–30) days in the infected group and 14 (4–18) days in the colonized group (*p* = 0.391).

Among colonized patients who did not develop infection, the most common site of colonization was the rectum 13/25 (53%), followed by urine 4/25 (16%). In the infected group 4/13 (31%) patients had a positive rectal swab and 3/13 (23%) had a positive sample from the respiratory tract prior to infection.

Twelve (92%) patients with documented NDM ABC infection received cefiderocol, and 10 (83%) patients in combination with colistin. Treatment was initiated before the availability of AST due to clinical severity. One patient was treated with ampicillin/sulbactam plus colistin due to history of cephalosporine anaphylaxis.

A microbiological cure was assessed in 9/11 patients with BSIs, 100% (9/9) demonstrated a rapid bacteria clearance with a median time from antibiotic initiation to first negative blood culture of 3 (1–4) days. 2/9 patients had a CVC-related BSI. Defining a microbiological cure in patients with osteomyelitis and VAP has not been possible.

A clinical cure on day 7 was achieved in 10 (83%) patients, one VAP, and 9 (82%) BSIs. Regarding those patients who did not achieve clinical cure at day 7: one patient was diagnosed with osteomyelitis, so clinical cure was not yet achieved at day 7; a second patient died at day 4 due to septic shock; a third patient had soft tissue infection after severe burn and subsequently developed another bloodstream infections sustained by Staphylococcus aureus preventing from defining clinical cure, though microbiological cure was achieved on day three. 

Median hospitalization was 94 (32–98) days with no difference between infected and non-infected patients, 94 (32–101) and 92 (23–91) days, respectively (*p* = 0.443). The fatality rate was 30% (4/13) in the infected group versus 4% (1/25) in the colonized group (*p* = 0.004). The only death in the colonized group has been related to underlying clinical conditions. 

Only two patients went through a cefiderocol monotherapy. The first one experienced osteomyelitis, the patient was exposed to 8 weeks of cefiderocol therapy with resolution of the infection. The second one had sepsis. Cefiderocol susceptibility test in disc diffusion, microdilution 1st and 2nd teste have been, respectively, 16 mm, 4 µg/mL and 2 µg/mL. The patients experienced partial response at 48 h and complete response at 7 days; hemoculture was confirmed negative at 72 h from antimicrobial therapy initiation. Unfortunately, the patient died 5 days after the end of cefiderocol therapy due to cardiological complications. 

### 2.3. Antimicrobial Susceptibility 

Out of 12 ACB NDM strains tested: 8/12 (67%) were collected from blood cultures, 3/12 (25%) from pharyngeal and rectal surveillance swabs, and 1 (8%) from bronchoalveolar lavage. All the NDM ABCs show a difficult-to-treat (DTR) phenotype, with preserved susceptibility only to colistin (Table 2). For three patients with surveillance swabs positive, ABC NDM was isolated in clinical samples subsequently.

According to these criteria, among 12 isolates 83% (10/12) were found to be resistant using disk diffusion, while 100% (12/12) and 75% (9/12) had a MIC value ≥ 2 mg/L with UMIC and ComASP gradient tests, respectively. 

Based on ECOFF, 10/12 and 12/12 isolates for disk diffusion and broth microdilution, respectively, fell above the ECOFF values, and thus were not wild-type strains.

## 3. Discussion

We show an effective strategy for treating an insidious pathogen like *A. baumannii* with an acquired NDM-1 enzyme. The association of cefiderocol plus colistin demonstrates a high clinical cure rate and a relatively low mortality rate compared to other reports [13]. *Acinetobacter baumannii* is a difficult-to-treat bacteria due to its high antibiotic resistance profile and nosocomial outbreaks involving, generally, severely ill patients [20]. 

Despite the high clinical cure reported in our experience, overall mortality remains considerably high, up to 30%. 

Few therapeutic options are available for CRAB and current international guidelines [9,10] recommend against the use of cefiderocol as first-line therapy, though no specific statement is made on MBL-expressing CRAB, which is not commonly encountered, differently from MBL-expressing carbapenem-resistant *Enterobacterales* [21].

Cefiderocol is a first-in-class siderophore cephalosporin that combines a catechol-type siderophore and cephalosporin core with side chains like cefepime and ceftazidime. This structure and its unique mechanism of action confer enhanced stability against hydrolysis by many β-lactamases, including extended-spectrum β-lactamases such as CTX-M, and Ambler’s class B and D carbapenemases such as KPC, NDM, VIM, IMP, OXA-23, OXA48-like, OXA-51-like and OXA-58 [22]. As currently suggested by IDSA and ESCMID guidelines, its use may be limited to the treatment of CRAB infections refractory to other antibiotics or in cases of intolerance or resistance to other agents. Nonetheless, retrospective studies showed better efficacy of cefiderocol in CRAB infections compared to randomized controlled trials [13,14]. However, retrospective analysis suffers from small sample sizes and the heterogeneity of treatments and subjects’ conditions [13,14]. Moreover, a higher rate of clinical or microbiological failure seems to be related to peculiar host conditions, such as clinical severity (i.e., septic shock, prolonged ICU stay, and mechanical ventilation) or strong immunosuppression [13,23].

Another critical issue for the use of cefiderocol in CRAB infections is related to susceptibility testing. According to CLSI [24] and EUCAST [25], the reference method for in vitro susceptibility of cefiderocol is both microbroth dilutions in iron-depleted, cation-adjusted Mueller–Hinton broth but the preparation of this medium is highly time-consuming and not appropriate for a daily workflow in a clinical microbiological laboratory. Moreover, the absence of a commercial standardized kit for broth microdilution susceptibility assay limits intra- and inter-laboratory reproducibility. Several commercial methods have been developed but issues in terms of accuracy, reproducibility, and bias have been detected discouraging their use [26].

IDSA guidelines [9] recommend, when cefiderocol is used, to carefully check sensitivity as recurrence and failure are more common in those where resistance occurs. International surveillance studies indicate that approximately 95% of CRAB isolates are susceptible to cefiderocol using the CLSI susceptibility criteria ≤4 µg/mL (Table 2) [27,28,29]. 

Unfortunately, obtaining reliable data about cefiderocol susceptibility is challenging, since hetero resistance or adaptive resistance, not detected by standard antimicrobial susceptibility testing methods, may be observed [30,31]. Even though their clinical relevance has not been demonstrated with a sufficient level of evidence [26], it has been hypothesized that hetero resistance might have contributed to cefiderocol treatment failure in the CREDIBLE-CR study [12].

In our experience, the in vitro activity of cefiderocol, theoretically the only beta-lactam that is potentially active against NDM-CRAB, does not appear to match in vivo effectiveness using currently available commercial tests. Noteworthy, all patients (9/9) with a BSI who repeated blood culture, after antimicrobial therapy had started, showed a rapid bacterial clearance (62 h, 48–72), even if 83%, 42%, and 50% of isolates were found resistant according, respectively, to disc diffusion susceptibility tests and the two microdilution methods, see Table 3. Cefiderocol plus colistin demonstrated a high microbiological cure rate regardless of the in vitro susceptibility test. In our experience in vitro tests demonstrated a low predictivity rate of in vivo cefiderocol activity.

A variety of mechanisms, typically acting in concert, have been reported to confer resistance to cefiderocol: β-lactamases (especially NDM, KPC and AmpC variants, OXA-427, and PER- and SHV-type ESBLs), porin mutations, and mutations affecting siderophore receptors, efflux pumps, and target (PBP-3) modifications. Co-expression of multiple β-lactamases, often in combination with permeability defects, appears to be the main mechanism of resistance [32]. NDM expression seems to facilitate the acquisition of resistance to cefiderocol even by additional mechanisms (such as mutations in siderophore receptors and increasing copy number of *bla_NDM_*) [33].

Among A. baumannii isolates, cefiderocol resistance prevalence was much higher among MBL-producing strains (40.9%, 95% CI 31.4–51.1%), especially in NDM-producers. Like other species, cefiderocol-non susceptibility prevalence was much lower when CLSI breakpoints were used compared to the EUCAST cut-off [32,33]. Moreover, NDM-like β-lactamases and, to a greater extent, PER-like β-lactamases were found to be associated with reduced susceptibility to cefiderocol in CRAB [34]. 

A recent study [35] has shown that on cefiderocol susceptible CRAB the most effective combination in terms of synergism and time-killing curves is represented cefiderocol plus tigecycline, while cefiderocol plus colistin—the main combination used in the treatment of NDM-CRAB in this series—show little synergism (47.7%). The same study demonstrated how the most active combination on cefiderocol-resistant CRAB is cefiderocol plus colistin, displaying the most potent bactericidal activity, with synergism in 100% case and no bacterial cells recovered within 24 h. In vivo an increased survival rate in the cefiderocol-colistin group in cefiderocol-resistant CRAB compared to cefiderocol and colistin monotherapy groups, with a 100% survival rate at 96 h. 

Since durlobactam has no activity on NDM [36,37], this new therapeutic option was not considered.

In our series, cefiderocol was combined with colistin in 10 patients, while in two cases colistin was not associated due to potential nephrotoxicity. A clinical cure was reached in 10 (77%) patients, which is higher than previously reported with other therapeutic regimens [38], and no relapse was observed [35]. By comparing to other observational reports, combination therapy (cefiderocol plus colistin) appeared to have a higher clinical cure rate than cefiderocol and colistin monotherapies, 66% and 44.4%, respectively [39].

With regards to the risk of clinical infection, it is noteworthy that only 54% (7/13) were colonized before infection. Conversely, 28% of colonized developed an infection in 8 [7,8,9,10,11,12,13] days. Compared to a previous report [40], a higher rate of infection has been observed among colonized patients and a shorter time from colonization to infection was documented, suggesting that NDM strains could carry higher pathogenicity.

The majority of patients have been treated with combination therapy. This choice was related to different elements: firstly, at the beginning of treatment we did not know the susceptibility test for cefiderocol, so, as monotherapy with colistin was not recommended [36], we started with a combination one; secondly, no other therapy options are available for this type of infection, so we decided to use an aggressive therapy from the very beginning. 

Unfortunately, we may not assess the specific role of every molecule in the resolution of the infection, but according to what we said before and the easy resistance development to colistin when used as a monotherapy [41], we may suppose cefiderocol plays a major role in the resolution of the infections.

Although international guidelines recommend against [9,10], Cefiderocol is gaining ground as a possible therapy for CRAB infections due to the limited treatment available at the moment.

Combination versus monotherapy is still an open debate. Lots of in vitro data and some in vivo studies confirm the synergistic effects of different combinations related to the genotype and phenotype of ABC [42]. Although, the relationship between combination therapy and better outcomes is still not clear in CRAB infections as well as other Gram- [43], some guidelines underlined that in case of complicated infections or MDR pathogens, combination therapy has to be considered [44].

Our study has several limitations. Firstly, it is a small retrospective single-center study, thus not leading to a generalization of the results. Secondly, we presented mainly bloodstream infections, with only one VAP, so the results may be carefully evaluated in this context. Moreover, the lack of genetic analysis may have prevented the assessment of further pathogenicity factors affecting the clinical course. Finally, the unavailability of reference methods for cefiderocol susceptibility tests, though common in clinical practice, limits the value of considerations regarding the agreement between in vitro activity and in vivo clinical efficacy.

Wider studies are needed to assess the best clinical approach in this subgroup of severe infections.

## 4. Methods

A single-center retrospective study including consecutive patients with infection or colonization by NDM ABC admitted at Niguarda Hospital in Milan from May 2022 to May 2023 was performed. 

All patients who tested positive for ACB NDM from rectal swabs, urine colonization, upper or lower respiratory tract, and groin swabs have been revised and included in the colonization group. All patients who developed an ACB NDM infection have been revised and included in the infection group.

Cefidercol has been used at 2 g every 8 h. Colistin has been used at 4.5 million/U every 12 h. 

Demographic, clinical, and biochemical data were collected from hospital electronic records. As a partial response to antimicrobial therapy, we define a response characterized by a reduction in the inflammatory index (white blood cells, C reactive protein, and procalcitonin) or apyrexia as a complete response reduction in the inflammatory index and apyrexia. ACB NDM has been isolated from as many patients in intensive care and burn unit. Species identification was performed by MALDI-TOF MS (BioMérieux, Sydney, Australia), NDM production was confirmed by immunochromatographic assay (NG-Biotech, Guipry-Messac, France), and antimicrobial susceptibility was evaluated by Microscan Walkaway Plus (Beckman Coulter, Brea, CA, USA) and Sensititre TM EURGGNCOL for colistin (Thermo Fisher Scientific, Waltham, MA, USA).

Of the 13 NDM ABCs involved in infections, 12 of them have been tested for cefiderocol susceptibility. In vitro cefiderocol activity was assessed by replicating the test with three commercially available methods: 2 microdilution panel (ComASP^®^, Liofilchem, and UMIC^®^, Bruker, Billerica, MA, USA) and 1 disk diffusion (30 µg) (Oxoid, Thermofisher Scientific) on Mueller Hinton II Agar (Liofilchem, Teramo, Italy) for each sample.

Infection and colonization were defined using US Centers for Disease Control and Prevention (CDC) standardized definitions study [45].

Since EUCAST has not yet established cefiderocol clinical breakpoints for Acinetobacter spp., the EUCAST PK/PD (non-species-related) tables of 2023 (EUCAST clinical breakpointtables v. 13.1) [25] were applied for the interpretation of MIC values, while for disk diffusion a zone diameter ≥17 mm was considered to correspond to MIC values below the PK-PD breakpoint of susceptibility (≤2 mg/L). Considering EUCAST epidemiological cut-off (ECOFF) [46] for the A. baumannii/cefiderocol association, ECOFF of 0.5 mg/L for MIC and 18 mm for disc diffusion was established (MIC and zone diameter distributions and ECOFFs). 

Based on CLSI guidelines (CLSI Performance Standards for Antimicrobial Susceptibility Testing 33rd ed. CLSI supplement M100. Wayne, PA: Clinical and Laboratory Standards Institute; 2023) [24] a microorganism with a MIC value ≤4 mg/L is considered susceptible, 8 mg/L intermediate and ≥16 mg/L resistant, while a strain with zone diameter ≥15 mm is considered susceptible. Disk diffusion zone diameters ≤14 mm should not be interpreted or reported since may occur with resistant, intermediate, or susceptible isolates, thus it is recommended to perform a MIC test.

Descriptive statistics (median and interquartile range [IQR] for continuous variables, and absolute and relative [%] values for categorical variables) were used to define the features of the study population. Non-parametric tests were applied to compare the groups: one-way ANOVA for independent measures for continuous and Fisher’s exact test for categorical variables. Two-tailed *p*-values were calculated and a value below 0.05 was considered statistically significant. Data management and analysis were performed using the STATA package, version 16.1 (StataCorp 2019, College Station, TX, USA).

## 5. Conclusions

CRAB is a relevant clinical issue for difficult-to-treat infections and the severity of the outcome. The acquisition of Ambler’s class B enzymes enhances these infections complexity. Cefiderocol plus colistin may be considered one of the possible therapeutical approaches in the setting of ACB NDM infections, though implementation of susceptibility tests and a better comprehension of the efficacy of cefiderocol in this context is essential. For sure infection control measures with systematic screening and isolation remain mandatory for the prevention of MDRO/DTRO diffusion and avoidance of outbreaks.

## Figures and Tables

**Table 1:** Clinical characteristics.

	Tot	Infection	Colonized	*p* Value
Patients, number (%)	38	13 (34%)	25 (66%)	
Age(y) median (IQR)	68 (55–74)	67 (56–77)	67 (57–76)	1.000
Male (N, %)	25 (66%)	8 (62%)	17 (68%)	0.730
Immunosuppressed (N, %) - Burns - Diabetes - Hematological malignancy - SOT - Connettivitis - HD - Cirrhosis - HIV	14 (40%)	7 (54%)	7 (28%)	
	6 (16%)	5 (40%)	1 (4%)	0.162
	3 (8%)	1 (8%)	2 (8%)	0.021
	3 (8%)	1 (8%)	2 (8%)	1.000
	1 (3%)		1 (4%)	1.000
	2 (5%)		2 (8%)	
	1 (3%)		1 (4%)	
	1 (3%)	1 (8%)		
	1 (3%)		1 (4%)	
Prior surgery	7(18%)	2 (15%)	5 (20%)	1.000
Charlson, median (IQR)	4 (2–5)	4 (2–5)	4 (2–5)	1.000
Previous antibacterials (N, %)	31 (82%)	11 (85%)	20 (80%)	1.000
ICU days, median (IQR)	16 (7–21)	21 (15–30)	14 (4–18)	0.391
In-hospital stay (median days)	94 (32–98)	94 (32–101)	92 (23–91)	0.433
Reason of in-hospital admission (N, %) - trauma - burns - septic shock - other infections - cardiovascular disease - neurological disease - renal disease				
	6 (16%)	2 (15%)	4 (16%)	1.000
	7 (18%)	4 (31%)	3 (12%)	0.202
	3 (8%)		3 (12%)	
	14 (36%)	5 (40%)	9 (36%)	1.000
	3 (8%)	1 (8%)	2 (8%)	1.000
	4 (10%)	1(8%)	3 (12%)	1.000
	1 (3%)		1 (4%)	
Isolation site (N, %) rectal swab urine upper respiratory tract BAL multiple colonization sites other				
	16 (42%)	3 (23%)	13(52%)	0.175
	4 (11%)		4 (16%)	
	6 (16%)	2 (15%)	4 (16%)	1.000
	1 (3%)	1 (8%)		
	2 (13%)	1 (8%)	1 (4%)	1.000
	3 (8%)		3 (12%)	
Positive blood culture (N, %) peripheral vein central vein	11 (29%)	11 (85%)		
	8 (21%)	8 (73%)		
	3 (8%)	3 (27%)		
Ventilator-associated pneumoniae (N, %)	1 (3%)	1 (8%)		
Time from first positive swab to infection days, median (IQR)	8 (7–13)	8 (7–13)		
Time from admission to first positive swab, median (IQR)	15 (6–28)	19 (5–23)	14 (5–31)	0.445
Time from admission to infection, median (IQR)	21 (6–27)	21 (6–27)		
Septic shock (N, %)	6 (16%)	6 (46%)		
SOFA, median (IQR)	7 (6–9)	7 (6–9)		
Antimicrobial Therapy (N, %)				
colistin	11 (30%)	11 (85%)		
cefiderocol	12 (32%)	12 (92%)		
ampicillin/sulbactam	2 (5%)	2 (15%)		
other	5 (13%)	5 (40%)		
CRRT (N, %)	2 (5%)	2 (15%)		
Antibacterial days, median (IQR)	9 (6–14)	12 (6–12)		
Clinical outcome (N, %)				
Clinical response at 48 h (N, %)				
None	3 (23%)	3 (23%)		
Partial response	6 (46%)	6 (46%)		
Complete	3 (23%)	3 (23%)		
Clinical response at 7 days (N, %)				
None	1 (8%)	1 (8%)		
Partial response	1 (8%)	1 (8%)		
Resolution	10 (77%)	10 (77%)		
Microbiological cure (N, %)	9 (24%)	9 (70%)		
Time from antibiotic start to first negative blood culture (N, %)	3 (1–4)	3 (1–4)		
Outcome (death) (N, %)	5 (13%)	4 (30%)	1 (4%)	0.004

Abbreviations: SOT: solid organ transplant; HD: hemodialysis; VAP: ventilator-associated pneumonia, BAL: bronco-alveolar lavage.

**Table 2:** Results of the three in vitro methods for cefiderocol susceptibility test of strains isolated from the 12 patients treated with cefiderocol.

N°	Disc Diffusion (mm)	Microdilution.1 (MIC µg/mL)	Microdilution.2 (MIC µg/mL)	Colistin (MIC µg/mL)
1	15	4	8	0.5
2	16	4	4	≤0.5
3	16	4	8	0.5
4	19	4	8	1
5	15	8	2	≤0.5
6	16	8	8	0.5
7	16	4	4	1
8	15	4	4	1
9	0	>32	32	1
10	16	16	16	0.5
11	19	8	2	1
12	16	4	2	0.5

Abbreviations: MIC: minimum inhibitory concentration.

**Table 3:** Correlation between in vitro methods for cefiderocol susceptibility test of strains isolated and microbiological and clinical response to the treatment.

N°	Disc Diffusion (mm)	Micro-Dilution.1 (MIC µg/mL)	Micro-Dilution.2 (MIC µg/mL)	Negative Blood Culture (hours)	Clinical Response, Complete (days)
1	15	4	8	48	Partial response at 7 days
2	16	4	4	24	2
3	16	4	8	72	2
4	19	4	8	Nd	7
5	15	8	2	72	7
6	16	8	8	48	Dead
7	16	4	4	VAP	7
8	15	4	4	72	2
9	0	>32	32	Nd	7
10	16	16	16	96	7
11	19	8	2	Osteomyelitis	Nd
12	16	4	2	72	7

Abbreviations: MIC: minimum inhibitory concentration; VAP: ventilator-associated pneumonia; Nd: no data.

## Data Availability

Data are available from the corresponding author upon reasonable request.
